# Supplementary material for: Time‑resolved multi-omic analysis of paclitaxel exposure in human iPSC‑derived sensory neurons unveils mechanisms of chemotherapy‑induced peripheral neuropathy
Source: Cell Death Dis. 2026 Feb 10;17(1):211. doi: 10.1038/s41419-026-08445-2 (PMC12921266; doi:10.1038/s41419-026-08445-2)
Supplement: Supplementary file 15 — Dataset 1. [file 41419_2026_8445_MOESM15_ESM.zip › CDD_Open_Data/04_RNA_Seq/Timeline_panel_plots_etc_2023-01-23_1609.zip!/Panel_Plots_etc/04_tmod_panel_plot_msigdb_reactome_pval_filter_0.01_AUC0.7_L2FC1.pdf]

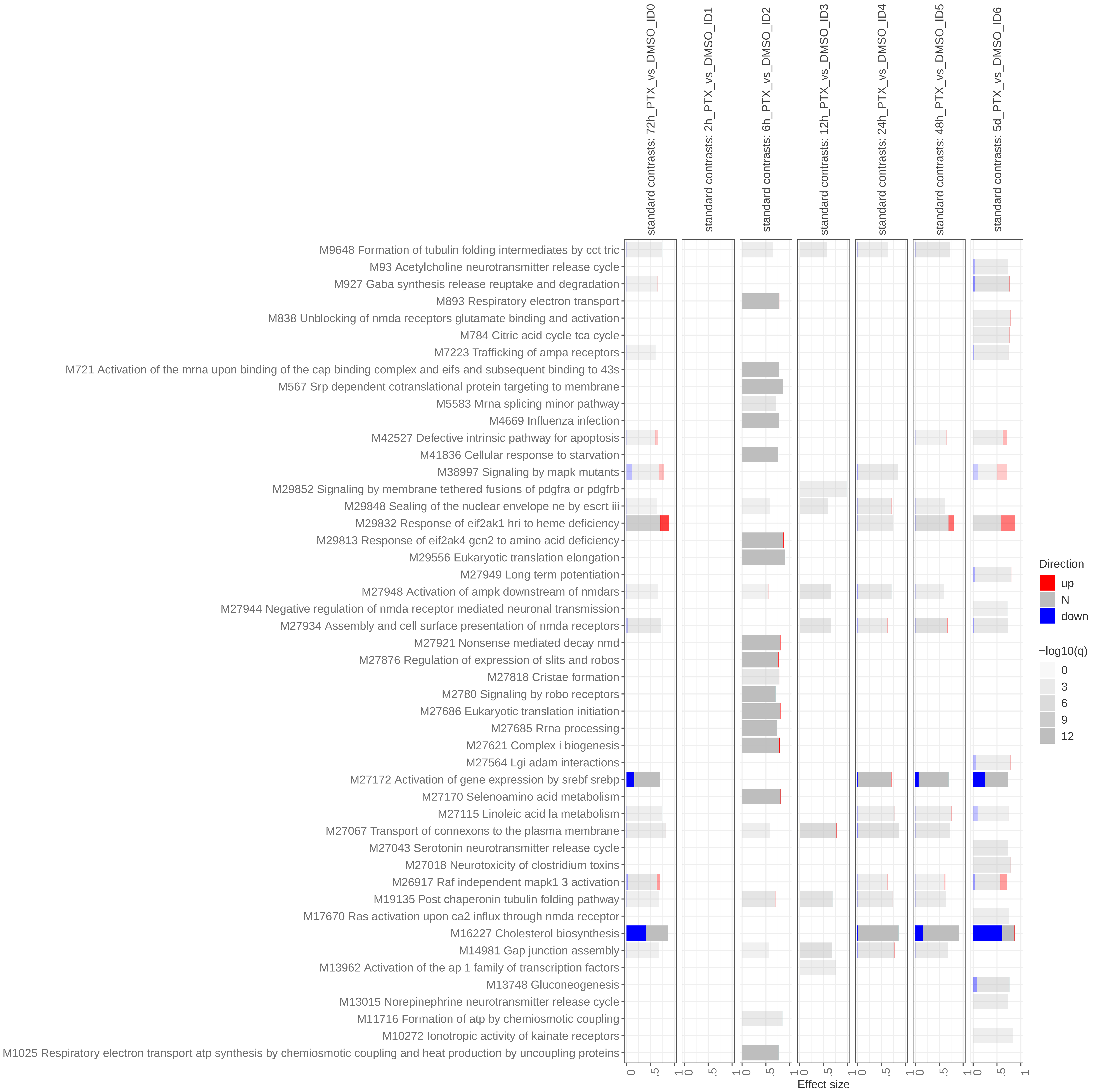

standard contrasts: 72h_PTX_vs_DMSO_ID0
standard contrasts: 2h_PTX_vs_DMSO_ID1
standard contrasts: 6h_PTX_vs_DMSO_ID2
standard contrasts: 12h_PTX_vs_DMSO_ID3
standard contrasts: 24h_PTX_vs_DMSO_ID4
standard contrasts: 48h_PTX_vs_DMSO_ID5
standard contrasts: 5d_PTX_vs_DMSO_ID6
M9648 Formation of tubulin folding intermediates by cct tric
M93 Acetylcholine neurotransmitter release cycle
M927 Gaba synthesis release reuptake and degradation
M893 Respiratory electron transport
M838 Unblocking of nmda receptors glutamate binding and activation
M784 Citric acid cycle tca cycle
M7223 Trafficking of ampa receptors
M721 Activation of the mrna upon binding of the cap binding complex and eifs and subsequent binding to 43s
M567 Srp dependent cotranslational protein targeting to membrane
M5583 Mrna splicing minor pathway
M4669 Influenza infection
M42527 Defective intrinsic pathway for apoptosis
M41836 Cellular response to starvation
M38997 Signaling by mapk mutants
M29852 Signaling by membrane tethered fusions of pdgfra or pdgfrb
M29848 Sealing of the nuclear envelope ne by escrt iii
M29832 Response of eif2ak1 hri to heme deficiency
M29813 Response of eif2ak4 gcn2 to amino acid deficiency
M29556 Eukaryotic translation elongation
M27949 Long term potentiation
M27948 Activation of ampk downstream of nmdars
M27944 Negative regulation of nmda receptor mediated neuronal transmission
M27934 Assembly and cell surface presentation of nmda receptors
M27921 Nonsense mediated decay nmd
M27876 Regulation of expression of slits and robos
M27818 Cristae formation
M2780 Signaling by robo receptors
M27686 Eukaryotic translation initiation
M27685 Rrna processing
M27621 Complex i biogenesis
M27564 Lgi adam interactions
M27172 Activation of gene expression by srebf srebp
M27170 Selenoamino acid metabolism
M27115 Linoleic acid la metabolism
M27067 Transport of connexons to the plasma membrane
M27043 Serotonin neurotransmitter release cycle
M27018 Neurotoxicity of clostridium toxins
M26917 Raf independent mapk1 3 activation
M19135 Post chaperonin tubulin folding pathway
M17670 Ras activation upon ca2 influx through nmda receptor
M16227 Cholesterol biosynthesis
M14981 Gap junction assembly
M13962 Activation of the ap 1 family of transcription factors
M13748 Gluconeogenesis
M13015 Norepinephrine neurotransmitter release cycle
M11716 Formation of atp by chemiosmotic coupling
M10272 Ionotropic activity of kainate receptors
M1025 Respiratory electron transport atp synthesis by chemiosmotic coupling and heat production by uncoupling proteins
0 .5 1
Effect size
Direction
up
N
down
−log10(q)
0
3
6
9
12
